# Stiffness Matters: Fine-Tuned Hydrogel Elasticity Alters Chondrogenic Redifferentiation

**DOI:** 10.3389/fbioe.2020.00373

**Published:** 2020-04-30

**Authors:** Barbara Bachmann, Sarah Spitz, Barbara Schädl, Andreas H. Teuschl, Heinz Redl, Sylvia Nürnberger, Peter Ertl

**Affiliations:** ^1^Faculty of Technical Chemistry, Institute of Applied Synthetic Chemistry and Institute of Chemical Technologies and Analytics, Vienna University of Technology, Vienna, Austria; ^2^AUVA Research Centre, Ludwig Boltzmann Institute for Experimental and Clinical Traumatology, Vienna, Austria; ^3^Competence Center MechanoBiology, Vienna, Austria; ^4^Austrian Cluster for Tissue Regeneration, Vienna, Austria; ^5^University Clinic of Dentistry, Medical University of Vienna, Vienna, Austria; ^6^Department Life Science Engineering, University of Applied Sciences Technikum Wien, Vienna, Austria; ^7^Division of Trauma-Surgery, Department of Orthopedics and Trauma-Surgery, Medical University of Vienna, Vienna, Austria

**Keywords:** cartilage, chondrocytes, 3D cell culture, extracellular matrix, hydrogel, Young’s modulus

## Abstract

Biomechanical cues such as shear stress, stretching, compression, and matrix elasticity are vital in the establishment of next generation physiological *in vitro* tissue models. Matrix elasticity, for instance, is known to guide stem cell differentiation, influence healing processes and modulate extracellular matrix (ECM) deposition needed for tissue development and maintenance. To better understand the biomechanical effect of matrix elasticity on the formation of articular cartilage analogs *in vitro*, this study aims at assessing the redifferentiation capacity of primary human chondrocytes in three different hydrogel matrices of predefined matrix elasticities. The hydrogel elasticities were chosen to represent a broad spectrum of tissue stiffness ranging from very soft tissues with a Young’s modulus of 1 kPa up to elasticities of 30 kPa, representative of the perichondral-space. In addition, the interplay of matrix elasticity and transforming growth factor beta-3 (TGF-β3) on the redifferentiation of primary human articular chondrocytes was studied by analyzing both qualitative (viability, morphology, histology) and quantitative (RT-qPCR, sGAG, DNA) parameters, crucial to the chondrotypic phenotype. Results show that fibrin hydrogels of 30 kPa Young’s modulus best guide chondrocyte redifferentiation resulting in a native-like morphology as well as induces the synthesis of physiologic ECM constituents such as glycosaminoglycans (sGAG) and collagen type II. This comprehensive study sheds light onto the mechanobiological impact of matrix elasticity on formation and maintenance of articular cartilage and thus represents a major step toward meeting the need for advanced *in vitro* tissue models to study both re- and degeneration of articular cartilage.

## Introduction

Articular cartilage is a specialized load-bearing tissue providing the ability of frictionless movement within synovial joints such as the knee, hip or shoulder. Generated by its single resident cell type, the chondrocyte, during embryogenesis the tissue is rearranged to withstand increasing mechanical forces until early adulthood (Carter et al., 2004). Upon reaching musculoskeletal maturity, the chondrocytes are embedded in a thick interwoven mesh of glycosaminoglycans and collagen type II. Adult articular cartilage is a relatively simple tissue with the extracellular matrix (ECM) forming up to 99% of the tissue while the chondrocytes cease their proliferative activity and acquire a metabolically quiescent state (Camarero-Espinosa et al., 2016). This characteristic simplicity, however, poses a major conundrum: given the absence of vascularization or innervation and the quiescent nature of chondrocytes, cartilage fails to regenerate if damaged by trauma or degenerative diseases (Hunziker et al., 2015). The relatively simple anatomy of cartilage – inhabitation by a single cell type and lack of vascular or nervous system – has led to the assumption that cartilage would be one of the first tissues to be successfully engineered (Temenoff and Mikos, 2000). Instead, primary chondrocyte isolation and cultivation proved to be inherently difficult, with a phenotype switch of primary chondrocytes during *in vitro* expansion posing a central challenge (Schnabel et al., 2002). The main indicators of this loss of differentiated phenotype are (i) an elongated morphology in contrast to chondrotypic sphericity, (ii) a switch from production of articular cartilage-specific collagen type II to fibrous tissue collagen type I and (iii) increased proliferative activity. The reversion into a differentiated phenotype can be achieved, by transferring chondrocytes into a three-dimensional (3D) culture setup within the first couple of passages after isolation (Caron et al., 2012). In other words, chondrocytes need to be cultivated as pellet, on a scaffold or suspended in a hydrogel to achieve chondrogenic behavior *in vitro*. Hydrogels, in particular, are ideally suited for 3D *in vitro* cultures owing to their ease of use, variety and flexibility. Hydrogels are hydrophilic polymeric networks capable of absorbing aqueous solutions multiple times their dry weight and thus present an ideal 3D cellular microenvironment emulating the native ECM characteristics of articular cartilage (Liu et al., 2017). These ECM-mimicking properties can further be exploited by the addition of chondrogenic molecules as well as by fine-tuning matrix elasticity. The elasticity of a matrix describes the resistance that cells feel in response to substrate deformation. Even though the elasticity of bodily tissues ranges from soft brain matter (1 kPa) to stiff collagenous, pre-calcified bone (100 kPa), its implication for *in vitro* tissue modeling is still widely unknown (Discher et al., 2009). While pilot studies in 2D culture substantiate the importance of matrix elasticity on cellular behavior (Engler et al., 2006), comprehensive studies conducted in a 3D environment remain scarce. Especially literature highlighting the influence of matrix elasticity on chondrocyte behavior is heterogenous and inconclusive. While some studies using monolayer cultures suggest that more compliant substrates foster the occurrence and maintenance of chondrogenic phenotypes (Schuh et al., 2010; Park et al., 2011), others mention surfaces exhibiting a cartilage-mimicking elasticity to provide chondroinductive effects (Allen et al., 2012; Du et al., 2016). In turn, the few comprehensive studies using 3D culture systems are challenging to interpret, since they lack comparability due to (a) the different methods used in determining and reporting matrix elasticity, (b) the choice of hydrogel type and elasticity ranges employed, and (c) used different chondrocyte passages, cultivation methods and cell sources (Schuh et al., 2012a; Vonwil et al., 2012). As a consequence of these methodological differences, beneficial effects of both soft, compliant substrates and stiff scaffolds emulating a chondrotypic matrix elasticity have been reported (Schuh et al., 2012b; Wang L. S. et al., 2014; Wang T. et al., 2014; Li et al., 2016). A short overview of current 3D studies investigating the interplay between matrix elasticity and chondrocyte behavior is provided in Table 1, further highlighting the heterogenicity of the state of the art.

**TABLE 1:** Overview of currently existing 3D chondrocyte models discussing matrix elasticity responses.

**Cell source**	**Passage**	**Endpoint**	**Hydrogel**	**Matrix stiffness**	**Modulus**	**Cell density**	**Outcome**	**References**
**Porcine**	P2	2 weeks	Agarose	0.75% (3.7 ± 1.9 kPa) 3.5% (53.2 ± 14.64 kPa)	Equilibrium	6*10^5^/mL	Increased proliferation in softer hydrogels No effect on chondrogenic phenotype	Schuh et al. (2012a)
**Rabbit**	P2	2 weeks	Gelatin-hydroxyphenylpropionic acid	570 Pa 1000 Pa 2750 Pa	Storage	1*10^6^/mL	Medium stiffness hydrogel exhibited superior 3D environment	Wang L. S. et al. (2014)
**Rabbit**	P2–P4	4 weeks	Chitosan-hyaluronic acid dialdehyde	130.78 ± 19.83 kPa 199.35 ± 81.57 kPa 181.47 ± 19.77 kPa	Young’s	5*10^6^/mL	Stiffer gels show spherical morphology and increased matrix synthesis	Thomas et al. (2017)
**Bovine**	P3	2 weeks	Gelatin- methacryloyl	3.8 ± 0.3 17.1 ± 2.4 29.9 ± 3.4 kPa	Young’s	2*10^7^/mL	Spherical morphology and enhanced matrix synthesis in highest stiffness	Li et al. (2016)
**Human**	P5	3 weeks	Gelatin - ethyl lysine diisocyanate	450 Pa 850 Pa	Shear	1*10^6^/construct	Softer gel more efficiently promoted chondrogenic differentiation	Sarem et al. (2018)
**Human**	P2	3 weeks	Fibrin Silk/Fibrin PEG-dextran	1 kPa 15 kPa 30 kPa	Young’s	1*10^6^/mL	High stiffness fibrin hydrogel induces spherical morphology and chondrogenic matrix synthesis	This study

**FIGURE 1:**
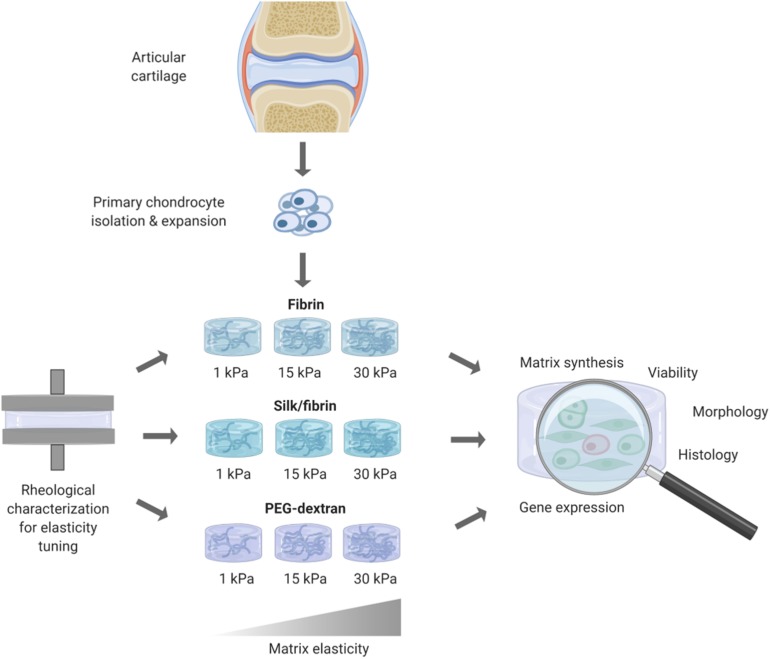
Overview of workflow for redifferentiation analysis of primary human chondrocytes in hydrogels of different elasticities. After rheological measurements for obtaining hydrogels of defined elasticities of 1 kPa, 15 kPa, and 30 kPa Youngs’s modulus, primary human chondrocytes where embedded and cultivated for 3 weeks. Chondrogenic redifferentiation was subsequently assessed by viability, histology and quantitative analysis of cartilage matrix components by GAG Assay and RT-qPCR.

In this work, the influence of matrix elasticity on primary human chondrocyte redifferentiation embedded in three different hydrogels was investigated in a comprehensive manner to determine the link between matrix elasticity and chondrogenic responses and close the knowledge gap associated with heterogenous prior reports. As visible in the graphical abstract in Figure 1, this was accomplished by embedding dedifferentiated human articular chondrocytes (hACs) in two natural hydrogels, fibrin and silk/fibrin, and one synthetic hydrogel, PEG-dextran. Silk fibroin has been added to further modulate matrix degradation and improve chondrogenic properties of fibrin hydrogels (Hofmann et al., 2006). While fibrin is an abundantly used (Hunter et al., 2004) protein-based biopolymer of natural origin that allows for cellular degradation and motility, PEG-dextran, in turn, is a chemically well-defined, highly biocompatible hydrogel that cannot be degraded by chondrocytes. In other words, bioinert PEG-dextran encapsulates chondrocytes resulting in physiologic shape control and movement restriction. Using oscillatory rheology, the matrix elasticity was adjusted to 1 kPa, 15 kPa, and 30 kPa Young’s modulus. The three chosen elasticity values represent the values of brain tissue, muscle fibers and that of the matrix surrounding each chondrocyte, the perichondral space, respectively (Guilak et al., 2005; Engler et al., 2006; Discher et al., 2009; Stolz et al., 2009). In addition to varying matrix type and elasticity, primary hACs were stimulated using TGF-β3 to gain insight into potential synergistic effects of matrix elasticity and growth factor presence. In summary, the aim of this study was the establishment of an improved articular cartilage *in vitro* model featuring spherical cell morphology, physiological gene expression as well as cartilaginous matrix deposition. It is envisioned that our improved *in vitro* model integrating biomechanics of articular cartilage can be employed in multiple cultivation systems including microbioreactors and organ-on-a-chip systems to study both re- and degenerative processes of the synovial joint.

## Materials and Methods

### Rheology

To assure that rheological characterization was performed within the linear-viscoelastic regime of each hydrogel as well as within the hydrogel’s respective low frequency plateau, rheometer settings were chosen in accordance with published protocols specifically designed for the rheological characterization of hydrogels (Zuidema et al., 2014). For the determination of Young’s Moduli, polymeric networks were assumed to display affine behavior. Affine models assume that within the deformation of the polymeric network the macroscopic strain equals the strain of each individual fiber (Wufsus et al., 2015). Rheological measurements were performed until desired hydrogel compositions displaying designated Young’s modulus values of 1 kPa, 15 kPa, and 30 kPa were ascertained.

Hydrogel-dependent gelation kinetics were studied using an MCR 302 WESP rheometer (Anton Paar, Austria), employing a parallel plate geometry (*d* = 25 mm, stainless steel) at 0.3 mm measuring gap distance. Hydrogel components were mixed thoroughly before injecting and subsequently recording dynamic oscillatory time sweeps [0.1% strain for fibrin (Zuidema et al., 2014) and 10% strain for PEG-dextran as determined by strain sweep shown in S1; 1 Hz] at 37°C. Hydrogel mixtures were applied to the precooled rheometer plate (4°C) to prevent premature polymerization and the measurement gap was sealed using paraffin oil to avoid sample evaporation. To guarantee reproducible data analysis, a simple mathematical evaluation protocol was established. Recorded data was analyzed by generating the first derivative thereof, calculating 10% of the curves slope, followed by averaging the Young’s modulus from the first 100 values were the slope falls below 10% of the peak value. The Young’s modulus was calculated by the following formula:

E= 2⁢G⁢(1+v)

where *E* is the Young’s modulus, *G* is the complex modulus, and *v* is the Poisson’s Ratio, accounting for the response in the directions orthogonal to the uniaxial stress. An affine network dominated by stretching modes was assumed and therefore a Poisson’s ratio of 0.5 was employed for the calculation of the Young’s modulus. Each measurement was performed in triplicates and the data represents the average thereof with the corresponding standard deviations.

### Primary Human Chondrocyte Isolation

Human articular chondrocytes were collected from the femoral head of three patients undergoing hip replacement, with patient’s consent and the approval of the ethical board of the Medical University of Vienna (No 1741/2018). Articular chondrocytes were isolated by 1 h incubation in 0.1% hyaluronidase solution (Sigma-Aldrich, Austria), 0.5 h in 0.1% pronase solution (Roche, Switzerland) followed by overnight incubation in a mixture of 200 U/mL collagenase (Gibco, United States) and 1 U/mL papain (Sigma-Aldrich, Austria). HAC were expanded in monolayer culture until passage two prior to hydrogel embedding. Medium was exchanged twice a week using chondrogenic expansion medium consisting of DMEM high glucose with 10% FCS, 1% antibiotic antimycotic solution, 10 mM HEPES, 6 mM L-glutamine, 5 μg/mL insulin, 50 μg/mL L-ascorbate-2-phosphate (all from Sigma-Aldrich, Austria).

### Hydrogel Embedding and 3D Chondrocyte Culture

For hydrogel embedding, primary human chondrocytes in passage 2 were detached from the culture flasks, pelleted and resuspended using a concentration of 4^∗^10^6^ cells/mL. Hydrogel clots were prepared according to compositions given in Table 2 and as per manufacturer’s instructions. Briefly, fibrin hydrogels were mixed to achieve final concentrations of 15 mg/mL, 27 mg/mL or 50 mg/mL fibrinogen (Tisseel, Baxter, Austria) and 1 U/mL thrombin (Tisseel, Baxter, Austria). Silk/fibrin hydrogels were prepared using the same fibrinogen and thrombin concentrations with the addition of 25% silk fibroin solution isolated from *Bombyx mori* as previously described (Teuschl et al., 2014). Silk fibroin was vortexed for 10 min at 3,000 rpm to induce β-sheet formation (Yucel et al., 2009) prior to mixing with cell suspension and fibrin hydrogel components. PEG-dextran hydrogels (Cellendes, Germany) were generated using 2.3 mM, 5 mM or 7.5 mM PEG-linker and 3 mM, 5.8 mM or 8.2 mM SG-dextran. All hydrogels were prepared in clots with 50 μL volume and a final cell concentration of 1,000 cells/μL hydrogel. Depending on the type of hydrogel, clots polymerized for 30 min up to 1 h before addition of cell culture medium. Hydrogel clots were cultivated over a period of 21 days with medium changes two times a week. The experiments were conducted using three individually prepared clots per analysis using chondrogenic differentiation medium consisting of DMEM with 1% antibiotic antimycotic solution, 1% L-glutamine, 1% HSA/linoleic acid mixture, 14 μg/mL L-ascorbate-2-phosphate, 1% ITS premix, 1 mM dexamethasone (all from Sigma-Aldrich, Austria) without or with the addition of 10 ng/mL TGF-b3 (R&D Systems, United Kingdom).

**TABLE 2:** Rheologically determined loss moduli, storage moduli as well as complex moduli, calculated Young‘s Moduli and concentrations of respective hydrogel components for fibrin and PEG-dextran hydrogels to achieve predefined elasticities of 1 kPa, 15 kPa and 30 kPa Young’s modulus.

	**Loss**	**Storage**	**Complex**	**Young’s**	**Average gelation**		
**Hydrogel**	**modulus [kPa]**	**modulus [kPa]**	**modulus [kPa]**	**modulus [kPa]**	**time [h]**	**Component 1**	**Component 2**
**Fibrin**	0.0182 ± 0.0067	0.37 ± 0.1	0.37 ± 0.1	1.1 ± 0.3	0.87	15 mg/mL Fibrinogen	1 U/mL Thrombin
	0.158 ± 0.095	4.6 ± 0.4	4.6 ± 0.4	13.8 ± 1.3	1.50	27 mg/mL Fibrinogen	1 U/mL Thrombin
	0.307 ± 0.0279	10.6 ± 1	10.6 ± 1	31.8 ± 2.8	1.67	50 mg/mL Fibrinogen	1 U/mL Thrombin
**PEG-Dextran**	0.0007 ± 0.0002	0.35 ± 0.1	0.35 ± 0.1	1.0 ± 0.3	5.1	2.3 mM PEG-Linker	3 mM SG-Dextran
	0.0018 ± 0.0032	5.4 ± 0.6	5.4 ± 0.6	16.2 ± 1.8	1.6	5 mM PEG-Linker	5.8 mM SG-Dextran
	0.0076 ± 0.0067	9.9 ± 1.0	9.9 ± 1.0	29.6 ± 3	1.5	7.5 mM PEG-Linker	8.2 mM SG-Dextran

### Live/Dead Assay

To obtain information on cellular vitality, a viability assay using calcein-AM and ethidium-H1 (Invitrogen, Austria) was performed according to the manufacturer’s instructions after a cultivation period of 21 days. Viability was assessed by comparing the number of live cells to total cell count.

### Histology

For paraffin histology, the samples were fixed in 4% buffered formalin for 24 h and subsequently rinsed in running tap water for 1 h. Afterward, the samples were transferred to 50% ethanol for 1 h and then stored in 70% ethanol until further processing. For paraffin embedding, the samples were first dehydrated in an uprising series of ethanol and then embedded in paraffin. The samples were cut with a Microm S in 4 μm sections and dried overnight in an incubator at 37°C. After removing the paraffin with xylene and rehydration the slides were stained histochemically with H&E or alcian blue at pH 2.5, which is ideal for the proof of glycosaminoglycans. For staining of collagen type II via immunohistochemistry, the slides were pretreated with pepsin (Sigma-Aldrich, Austria) for 10 min in a humidity chamber at 37°C. Subsequently, endogenous peroxidase and alkaline phosphatase were blocked using the ready-to-use BloxAll (Vector Laboratories, United States) solution for 10 min. Afterward, the slides were blocked with 2.5% normal horse serum (Vector Laboratories, United States). Then the slides were incubated with collagen type II antibody (Neomarkers, United States), 1:100 for 1 h in the Labvision Autostainer 360 (Thermo Scientific, United States). The secondary HRP-anti-mouse antibody (ImmunoLogic, Netherlands) was incubated for 30 min and then the sections were treated with the ImmPACT NovaRed (Vector Laboratories, United States) peroxidase substrate for 6 min. After rinsing the slides in water they were counterstained with hematoxylin, dehydrated and permanently embedded with Consul-mount (Thermo Scientific, Waltham, United States).

### RT-qPCR

For gene expression analysis, hydrogel clots were enzymatically digested by adding either 100 U/mL nattokinase solution (Japan Bio Science Laboratory Co., Ltd., Osaka, Japan) for fibrin and silk/fibrin hydrogels (Heher et al., 2015) or a 1:20 dilution of dextranase (Cellendes, Germany) for PEG-dextran hydrogels as per the manufacturer’s instructions and subsequently incubated at 37°C for 1 h. Thereafter the digested clots were snap frozen by submersion into liquid nitrogen and stored at −80°C up to the point of analysis. RNA was extracted using TRIzol reagent (Invitrogen, Austria). Total extracted RNA was quantified using a NanoDrop ND-1000 spectrophotometer (Thermo Fisher Scientific Inc., United States) at 260/280 nm and DNA was removed by employing the RQ1 RNAse-Free DNAse Kit (Promega, Austria). Next, complementary DNA (cDNA) was synthesized from 2 μg total RNA employing the OneScript^TM^ cDNA Synthesis Kit (ABM) according to the manufacturers’ instructions. Finally, RT-qPCR analysis was accomplished using a Bio-Rad C1000/CFX Cycler as well as the KAPA SYBR Fast Kit (VWR, Austria). To assess both primer quality (see Table 3) and required primer concentration an initial standardization was performed using purified total RNA of primary hACs. Fold change in gene expression was calculated by the 2^–ΔΔ*CT*^ formula relative to the housekeeping gene B2M and referenced to same-passage chondrocytes cultivated in monolayer.

**TABLE 3:** Primers employed in gene expression analysis with RT-qPCR.

**Gene**	**Denotation**	**Characteristics**	**Forward primer**	**Reverse primer**
**ACAN**	Aggrecan	ECM component	TCSGAGGACA GCSGAGGCC	TCSGAGGGTGTA GCGTGTASGAGA
**Col1**	Collagen type I	Dedifferentiation	AGGTGCTGA TGGCTCTCCT	GGACCACTTT CACCCTTGT
**Col2**	Collagen type II	ECM component	CCACGCTCAA GTCCCTCAAC	AGTCACCGCT CTTCCACTCG
**B2M**	Beta micro-globulin 2	Housekeeping gene	ACTGAATTCA CCCCCACTGA	CCTCCATGAT GCTGCTTACA

### Glycosaminoglycan Quantification

For quantification of glycosaminoglycans, a standard protocol based on dimethyl-methylene blue (DMMB) precipitation was performed. Prior to the assay, PEG-dextran hydrogels were digested using dextranase (Cellendes, Germany) while no pretreatment was necessary for fibrin and silk/fibrin hydrogels. Briefly, samples were digested using 30 U/mL proteinase K (Sigma-Aldrich, Austria) at 56°C overnight before addition of DMMB (Sigma-Aldrich, Austria) reagent. Precipitates were centrifuged and the pellet was dissociated using decomplexion solution. Subsequently, absorption was measured at 560 nm within a PerkinElmer Ensight^TM^ multimode plate reader. A standard curve was prepared by the dilution of chondroitin sulfate (Sigma-Aldrich, Austria) in phosphate buffered EDTA/cysteine solution.

### DNA Quantification

Sample DNA was quantified using a DNA Quantitation Kit Fluorescence Assay (Sigma-Aldrich, Austria) as per the manufacturers’ instructions. Briefly, 10 μL of the proteinase K digest was mixed with 200 μL of bisbenzimide H33258 dye. Samples were measured in triplicates using a PerkinElmer Ensight^TM^ multimode plate reader.

### Data Analysis

Data was analyzed using GraphPad Prism software. ROUT outlier test was performed on all data sets and significant outliers (*Q* = 1%) were removed if applicable. Two-way ANOVA as well as Tukey’s multiple comparisons test were performed to compare the data sets.

## Results and Discussion

### Rheological Characterization of Hydrogels

Fine-tuning of matrix elasticity toward the desired Young’s moduli of 1 kPa, 15 kPa, and 30 kPa for fibrin and PEG-dextran was achieved using oscillatory rheology. Intended elasticity values represent a broad spectrum of matrix stiffness ranging from very soft tissues (e.g., brain tissue), to tissues with medium elasticity (e.g., muscle tissue) up to matrices with high elasticities which mimic the native pericellular microenvironment of the human chondrocyte (Discher et al., 2009). Results of rheological characterization of fibrin and PEG-dextran are listed in Table 2 featuring loss, storage and complex as well as Young’s moduli at the determined hydrogel compositions. Since preliminary rheological assessment of silk fibroin revealed a Young’s Modulus below 1 kPa (data not shown), the impact on matrix elasticity of 25% silk fibroin addition to fibrin hydrogels was considered negligible. For fibrin hydrogels, an increase in both storage and loss modulus as well as in Young’s modulus was achieved when incrementing total fibrinogen concentration at a constant thrombin concentration of 1 U/mL. Exemplary graphs of hydrogel gelation in Supplementary Figures S1, S2 show that the storage modulus contributes to a much higher extent to the complex modulus in comparison to the loss modulus, a characteristic behavior for hydrogels suggesting high intrinsic solidity. All examined parameters display linearity along the range of increasing stiffness with Young’s moduli ranging from 1.1 ± 0.3 kPa for hydrogels with 15 mg/mL fibrinogen to 13.8 ± 1.3 kPa for hydrogels with 27 mg/mL fibrinogen up to 31.8 ± 2.8 kPa for hydrogels using 50 mg/mL fibrinogen. Overall, hydrogel polymerization was completed within 1–2 h, depending on the hydrogel’s stiffness. Similar to the results obtained for fibrin hydrogels, an increase in the Young’s moduli of PEG-dextran hydrogels was achieved when increasing both dextran and PEG-linker concentration accordingly, as depicted in Table 2. Concentrations of the PEG-linker and dextran ranged from 2.3 mM and 3 mM to attain a PEG-dextran hydrogel of 1.0 ± 0.3 kPa Young’s modulus to 5 mM and 5.8 mM for hydrogels of 16.2 ± 1.8 kPa Young’s modulus up to 7.5 mM and 8.2 mM for the highest elasticity hydrogels of 29.6 ± 3 kPa Young’s modulus. As visible in the gelation plots shown in Supplementary Figure S1A, PEG-dextran hydrogels displayed low loss moduli ranging from 0.7 ± 0.2 to 7.6 ± 6.7 Pa coupled with long gelation times of up to 5.1 h. Overall, both fibrin and PEG-dextran hydrogels proved to be well suited for fine-tuning of matrix elasticity.

### Hydrogel Type and Elasticity Affect Cell Viability and Morphology

**FIGURE 2:**
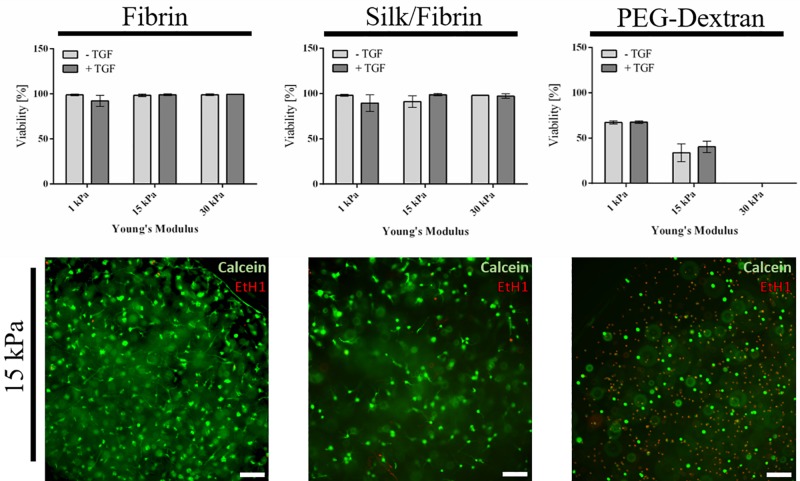
Viability of primary chondrocytes in fibrin, silk fibrin and PEG-dextran hydrogels depicted as mean with SD (*n* = 9). Fibrin and silk/fibrin hydrogels display high viabilities irrespective of stiffness while viability in PEG-dextran hydrogels declines rapidly with increasing elasticity. Epifluorescent exemplary images of Live(Calcein-AM)/Dead staining (Ethidium-H1) in 15 kPa hydrogels without growth factor addition (below). Scale bar 100 μm.

As a first qualitative evaluation of hydrogel-embedded chondrocyte behavior, cellular viability, and morphology were assessed using a calcein-AM-based viability assay after a cultivation time of three weeks. Images depicted in Figure 2 and Supplementary Figure S3 showing epifluorescence pictures of the whole hydrogel clot reveal that chondrocyte cultivation in both fibrin and silk/fibrin hydrogels resulted in high cell viabilities irrespective of hydrogel elasticity and growth factor presence. Despite high cell survival in both hydrogels, variations in cell density indicate a both hydrogel and matrix elasticity-dependent response in cell proliferation. For fibrin hydrogels, viability was consistently high with values ranging from 92 ± 6% in 1 kPa hydrogels with TGF-β3 to 99% ± 1% in 30 kPa hydrogels with TGF-β3. Similarly, high viabilities were observed in silk/fibrin hydrogels with values ranging from 89 ± 9% in hydrogels with 1 kPa elasticity with TGF-β3 to 98 ± 1% in 30 kPa hydrogels with TGF-β3. In contrast to these observations, chondrocytes embedded in PEG-dextran hydrogels showed significantly reduced viability as shown in the right panel of Figure 2. The viability values steeply declined from 67 ± 1% in 1 kPa hydrogels to no measurable viability in PEG-dextran hydrogels with an elasticity of 30 kPa Young’s modulus. This decline in viability may be a result of (a) a decrease in pore size (below 10 nm) with increasing crosslinking density and/or (b) increased cellular encapsulation restricting any cellular motility. The lower panel of Figure 2 shows exemplary images of chondrocytes embedded in fibrin, silk/fibrin and PEG-dextran hydrogel of 15 kPa Young’s modulus without growth factor addition. For a more comprehensive analysis on morphological changes induced by matrix elasticity, Supplementary Figure S3 shows images of live/dead stained chondrocytes in all three hydrogels of 1 kPa, 15 kPa, and 30 kPa matrix elasticity without and with growth factor supplementation. Comparable trends in cellular morphology were observed in both fibrin and silk/fibrin hydrogels, as visible in the upper panels of Supplementary Figures S3A,B. Importantly, chondrocyte sphericity increased while overall cell density decreased with increasing matrix elasticity, indicating a redifferentiated chondrogenic phenotype. Growth factor stimulation with 10 ng/mL TGF-β3 resulted in a more elongated morphology of embedded chondrocytes compared to their non-stimulated counterparts irrespective of matrix elasticity as also described by Lin et al. (2006). While chondrocytes cultivated in all matrix elasticities formed cellular protrusions, the general cell morphology was more elongated in hydrogels of 1 kPa and 15 kPa Young’s modulus compared to hydrogels of 30 kPa Young’s modulus, where chondrocytes retained a chondrotypic sphericity. In silk/fibrin hydrogels, however, the morphological changes with increasing elasticity were less distinct. In the presence of TGF-β3 stimulation chondrocytes organized into clusters, predominantly observed in 30 kPa hydrogels as could additionally be observed in histological analysis discussed in section “Increasing Matrix Elasticity Modulates Chondrotypic Morphology and Matrix Deposition.” This cluster formation can be explained by a combination of growth factor induced proliferation with a stiffness dependent restriction in cell motility. PEG-dextran hydrogels were specifically chosen to emulate chondrocyte encapsulation similar to widely used agarose and alginate hydrogels while providing matrix tunability and ease-of-use. Even though RGD-peptide was added to PEG-dextran hydrogel to increase hydrogel comparability and ameliorate a possible negative impact of lacking adhesion sites on cell behavior as recommended by the supplier, PEG-dextran hydrogel impaired cellular viability as depicted in Supplementary Figure S3C. Interestingly, chondrocytes encapsulated in 15 kPa and 30 kPa hydrogels exhibited a duality in live/dead staining in addition to low cell viabilities as shown in Figure 2 and Supplementary Figure S3C. This behavior, characterized by a red-stained nucleus and green-stained cytoplasm, suggests a hydrogel-inflicted permeability of the cellular membrane. These observations reveal the negative effect of PEG-dextran hydrogels with increasing elasticity on cell behavior. In conclusion, while chondrogenic morphology was supported by high matrix elasticity in the case of both fibrin-based hydrogels, undegradable PEG-dextran hydrogels promoted a spherical morphology in all elasticities but negatively affected cell vitality.

### Increasing Matrix Elasticity Modulates Chondrotypic Morphology and Matrix Deposition

**FIGURE 3:**
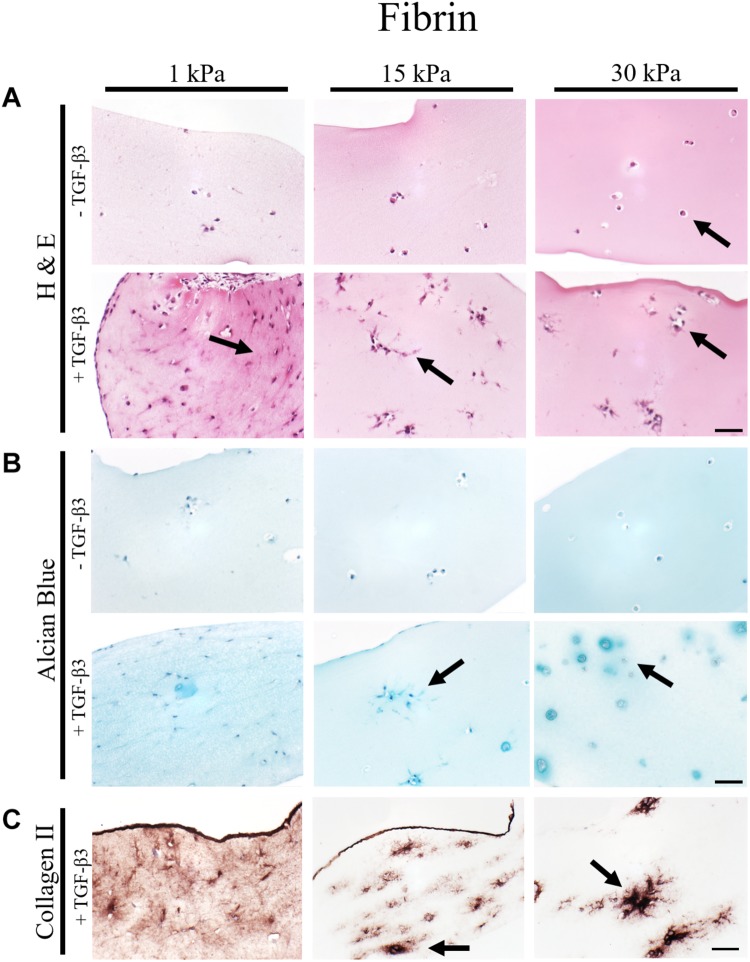
Histological images of 4 μm thick sections of primary chondrocytes in fibrin hydrogels of 1 kPa, 15 kPa, and 30 kPa elasticity stained with **(A)** H&E for morphology, **(B)** alcian blue for sGAG and **(C)** collagen II. Scale bar 50 μm. Arrows highlight visible morphological differences in the presence of increasing matrix elasticity and growth factor stimulation, where elongated, fibroblastic morphology is found in softer hydrogels, and chondrotypic spherical morphology in 30 kPa hydrogels. Growth factor stimulation resulted in an overall increase in cell numbers with stiffness-depended cell distribution including cellular cluster formations in 30 kPa hydrogels.

**FIGURE 4:**
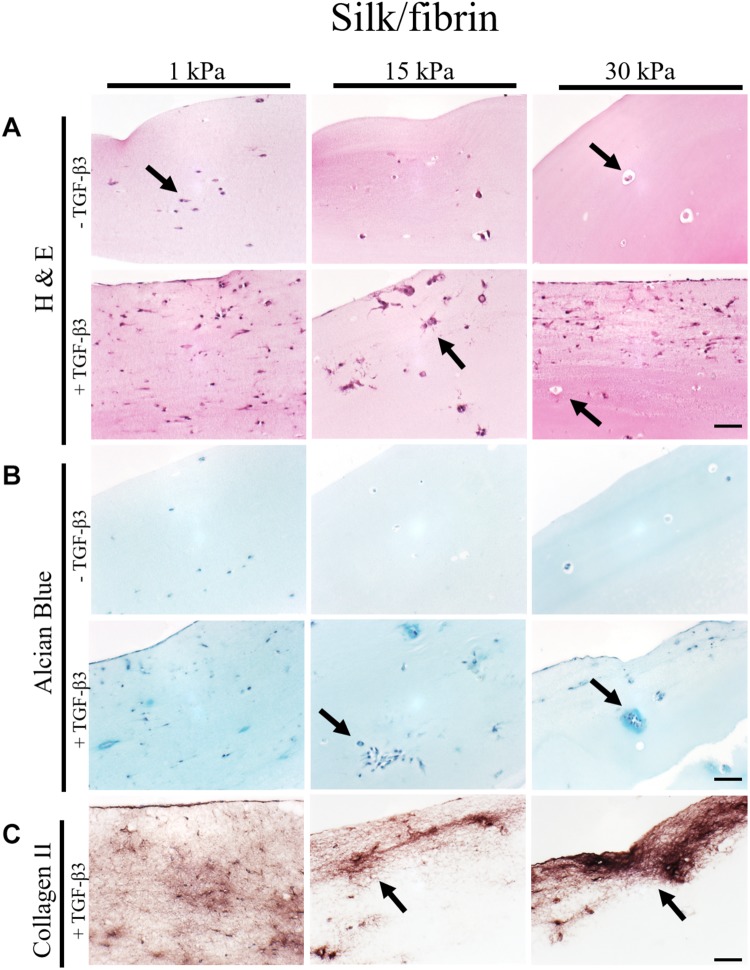
Histological images of 4 μm thick sections primary chondrocytes in silk/fibrin hydrogels of 1 kPa, 15 kPa and 30 kPa elasticity stained with **(A)** H&E for morphology, **(B)** alcian blue for sGAG and **(C)** collagen II. Scale bar 50 μm. Arrows highlight visible in the presence of increasing matrix elasticity and growth factor stimulation, resulting in enhanced chondrotypic sphericity with increasing hydrogel stiffness and cell cluster formation at 30 kPa hydrogels. Additional stimulation with growth factor resulted in massive formation of cartilage matrix collagen II around the clusters.

To gain deeper insights into morphological changes and ECM synthesis in response to increasing matrix elasticity, histological analysis of 4 μm thin sections was conducted. While H&E staining allows for the assessment of cellular morphology, alcian blue and collagen type II staining were performed to identify *de novo* synthesis of GAG and collagen type II by the embedded chondrocytes. Representative images of histological sections are shown in Figure 3 for fibrin hydrogel, Figure 4 for silk/fibrin hydrogel as well as in Supplementary Figure S4 for PEG-dextran hydrogels. Overall, results of histological analysis demonstrate a morphological trend toward increased number of fully spherical chondrocytes in the presence of high matrix elasticities. H&E staining of fibrin hydrogels, depicted in Figure 3A, shows that while a large fraction of chondrocytes exhibited a dedifferentiated elongated or polygonal morphology in hydrogels of 1 kPa and 15 kPa elasticity, almost all chondrocytes displayed a physiologic round morphology in 30 kPa hydrogels. Furthermore, stimulation with TGF-β3 seems to result in an increased cell density in all hydrogels, suggesting an elevated proliferation rate. While this growth-factor induced proliferation resulted in an even distribution of chondrocytes with dedifferentiated fibroblastic morphology in hydrogels with the lowest elasticity, chondrocytes embedded in 15 kPa and 30 kPa hydrogels formed small cellular clusters. Especially, chondrocytes in 30 kPa hydrogels were found to be densely packed and formed circular clusters, underlining a restricted cellular motility at increased matrix stiffness.

To assess the effect of hydrogel elasticity on matrix deposition, histological sections were stained with alcian blue to highlight sulfated sGAG, one of the key components of the native ECM. Figure 3B shows an increased presence of sGAG with increasing matrix stiffness only when stimulated with TGF-β3. Differences in sGAG deposition between hydrogels of low, medium and high elasticities can be associated with varying matrix distributions. Particularly striking was the formation of small alcian blue-positive halos around many spherical chondrocytes in fibrin hydrogels of the highest matrix elasticity. The presence of these halos resembles the pericellular and interterritorial matrix in chondrons, a characteristic feature of native cartilage tissue. In contrast to this chondrotypic behavior in 30 kPa hydrogels, sGAG staining was evenly spread throughout the hydrogel in fibrin hydrogels with the lowest matrix elasticity of 1 kPa Young’s modulus. To confirm physiological behavior within hydrogels of increasing matrix elasticity, immunohistochemical staining of the most common cartilaginous matrix protein, collagen type II, was performed. Results in Figure 3C reveal fibrillar collagen type II staining loosely distributed throughout the whole hydrogel at 1 kPa Young’s modulus and in-between cell clusters in 15 kPa hydrogels. In 30 kPa hydrogels collagen type II fibrils are co-localized with the alcian blue staining and densely packed around the chondrocyte clusters. In other words, chondrocytes embed themselves within a thick, native ECM-enriched microenvironment only when exposed to physiologic matrix elasticities.

In the case of silk modified fibrin hydrogels, shown in Figure 4, similar trends in chondrogenic morphology and native ECM deposition were observed with increasing matrix elasticity. Remarkably, the formation of small, chondron-like halos around each spherical chondrocyte in hydrogels with an elasticity of 30 kPa was also visible in this blended fibrin hydrogel (arrow Figure 4A). SGAG distribution in higher elasticity matrices shows striking similarity to *in vivo* cartilage, highlighting the positive effect of perichondral space-like matrix stiffness mimicked by 30kPa silk/fibrin hydrogels. Similar to observations in fibrin hydrogels, chondrocytes stimulated with TGF-β3 in 1 kPa silk/fibrin hydrogels exhibited a dedifferentiated fibroblastic morphology. The beneficial effect of TGF-β3 stimulation on ECM synthesis is demonstrated in Figures 4B,C, where sGAG and collagen type II production are clearly visible. Location of sGAGs as well as collagen type II again coincided with cellular distribution. In 1 kPa Young’s modulus hydrogels, both chondrocytes and ECM molecules were evenly distributed throughout the whole hydrogel clot. In contrast, sGAG secretion was mainly allocated with single spherical chondrocytes in silk/fibrin hydrogels of 15 kPa Young’s modulus or with chondrocyte clusters in 30 kPa Young’s modulus hydrogels. Overall, collagen type II was most pronounced in 30 kPa silk/fibrin hydrogels where chondrocytes generated a thick collagen type II matrix. Final histological analysis of chondrocytes embedded in PEG-dextran hydrogels showed no obvious differences in morphology as the synthetic, undegradable hydrogel encapsulates the cells in a spherical morphology. Additionally, Supplementary Figure S4 shows no increase in cell number despite TGF-β3 stimulation pointing at a hydrogel-associated restriction disenabling cellular motility. Confirming previous findings of reduced cellular viability, histological sections depict cellular debris and empty hydrogel pockets.

In summary, fibrin and silk/fibrin hydrogels with elasticities of 30 kPa resembling the stiffness of the perichondral space most effectively support chondrogenic redifferentiation based on spherical morphology and synthesis of native sulfated glycosaminoglycans as well as collagen type II. In contrast, chondrocytes embedded in elasticities resembling brain tissue exhibited a dedifferentiated elongated morphology but still synthesized cartilage-associated ECM molecules.

### Quantitative Analysis of Matrix Components

**FIGURE 5:**
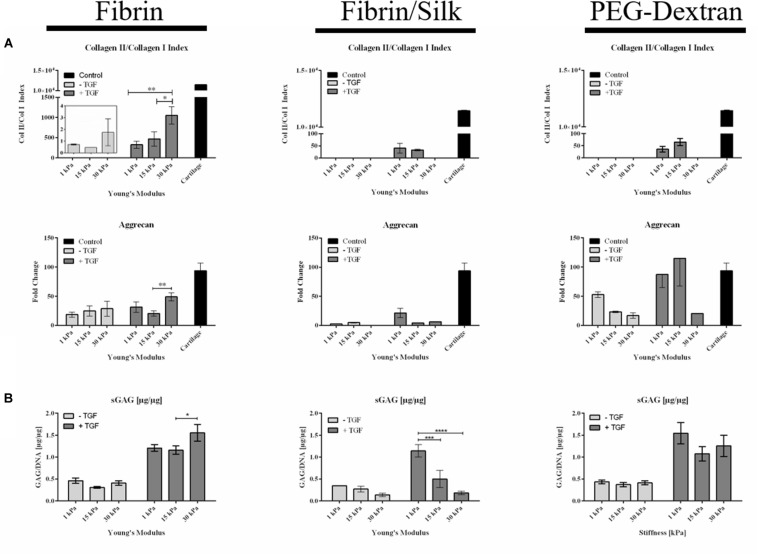
**(A)** Gene expression of redifferentiation indices and aggrecan expression in the presence of increasing hydrogel stiffness, resulting in incremental increases of collagen type II and aggrecan synthesis using fibrin hydrogels. In turn, in the presence of PEG-dextran hydrogels native amount of aggrecan synthesis was observed (results should be interpreted with caution due to low cell vitality). **(B)** Quantification of synthesized sGAG per μg DNA in fibrin, silk/fibrin and PEG-dextran hydrogel of varied elasticity again showed an improved redifferentiation behavior in fibrin hydrogels with increasing matrix elasticity. All results depicted as mean with SEM. *n* = 9; *p < 0.1; **p < 0.05; ***p < 0.01; ****p < 0.001.

To emphasize the qualitative observations from histological analysis, redifferentiation of primary human chondrocytes was assessed quantitatively using both RT-qPCR of collagen type I, collagen type II as well as aggrecan and glycosaminoglycan quantification. Redifferentiation indices in Figure 5 were calculated using the expression profiles of cartilaginous collagen type II in relation to dedifferentiated collagen type I. Individual gene expression changes are displayed in Supplementary Figure S5. Additionally, redifferentiation was evaluated based on the expression of aggrecan, an integral part of the cartilaginous matrix. In unstimulated fibrin hydrogels (left panel of Figure 5A) a minor increase in the redifferentiation index from 0.71 ± 0.05 in the softest hydrogels of 1 kPa Young’s modulus to 1.75 ± 1.15 in fibrin hydrogels of 30 kPa Young’s modulus was observed. A more prominent difference, however, was discernible in TGF-β3 stimulated cultures, where chondrocyte redifferentiation significantly increased with incremental changes in matrix elasticity. Here, redifferentiation indices ranged from 324 ± 88 for fibrin hydrogels of 1 kPa Young’s modulus to 470 ± 177 for 15 kPa hydrogels up to 1051 ± 212 for 30 kPa hydrogels resembling the elasticity of the native perichondral environment. It is important to note that the addition of TGF-β3 resulted in a 600-fold increase in the redifferentiation index, confirming the synergistic effects of growth factor TGF-β3 and native elasticity. In turn, the observed upregulation of collagen type I (see Supplementary Figure S5) in soft hydrogels clearly points at an undesired fibrocartilaginous response. The outcome that fibrin hydrogels of 30 kPa elasticity ideally support the differentiation process of primary human chondrocytes is additionally supported by an increase in aggrecan expression in TGF-ß3-unstimulated as well as in stimulated cultures as displayed in Figure 5A (lower panel). Fold change values were slightly elevated from 18.5 ± 4.4 in 1 kPa hydrogels to 28.6 ± 13.0 in 30 kPa hydrogels for unstimulated cultures and significantly increased from 20.5 ± 4.5 in 15 kPa hydrogels to 49.0 ± 7.0 in 30 kPa hydrogels following TGF-β3 stimulation. A similar correlation was observed for sGAG synthesis as results shown in Figure 5B (left panel) revealed the highest sGAG content of 1.55 ± 0.19 μg sGAG/μg DNA for TGF-β3 treated fibrin hydrogels of 30 kPa matrix elasticity. In comparison, 1.21 ± 0.08 μg sGAG/μg DNA was detected in 1 kPa hydrogels and 1.16 ± 0.1 μg sGAG were synthesized per μg DNA in hydrogels of 15 kPa matrix elasticity. Interestingly, the addition of 25% silk fibroin to the fibrin hydrogel negatively affected the expression of cartilaginous proteins as visible in the middle panel of Figure 5. While collagen type II expression in hydrogels without growth factor stimulation remained below the limit of detection, collagen type I expression was downregulated in the absence and presence of TGF-β3 with increasing matrix elasticity as depicted in the middle panel of Figure 5A and Supplementary Figure S5 (middle panel). For the remaining elasticities (e.g., 1 kPa and 15 kPa), the redifferentiation indices were almost 10-fold decreased compared to pure fibrin hydrogels with values of 40.2 ± 19.6 and 32.9 ± 3.6 for 1 kPa and 15 kPa hydrogels, respectively. Remarkably, a reverse trend was observed for aggrecan expression and sGAG synthesis, where ECM production was lowest with highest hydrogel elasticity. For aggrecan synthesis, this relates to a fold change of 21.3 ± 7.9 in 1 kPa hydrogels and 4.2 ± 0.4 in 15 kPa hydrogels. This upregulation in aggrecan expression and increased synthesis of sulfated glycosaminoglycans in the softest 1 kPa silk/fibrin hydrogels as shown in Figure 5B may be attributed to miscibility issues in higher stiffness hydrogels. The right panel in Figure 5 displays gene expression and sGAG synthesis profiles in PEG-dextran hydrogels. Despite limited cellular viability, redifferentiation indices incrementally increased with increasing matrix elasticity for TGF-β3 supplemented hydrogels, with mean index values of 35.5 ± 12.0, 65.6 ± 14.6 for 1 kPa and 15 kPa hydrogels, respectively. For PEG-dextran hydrogels of 30 kPa elasticity, however, no gene expression activity of collagen type I or collagen type II could be detected, probably owed to the limited chondrocyte viability in these hydrogels. Correspondingly, gene expression results related to 15 kPa and 30 kPa hydrogels must be interpreted with caution due to the small amounts of surviving cells within said hydrogels. Similar trends of declining ECM synthesis with the loss of cell vitality were seen in gene expression levels of aggrecan as well as sGAG content. However, aggrecan expression was slightly elevated in TGF-β3 stimulated PEG-dextran hydrogels of 15 kPa elasticity with a value of 115 ± 47 compared to 87.3 ± 22.3 in 1 kPa hydrogels. This means that the gene expression of aggrecan is comparable to levels in native cartilage controls, pointing at a positive chondrogenic effect of the PEG-dextran encapsulation. Additional quantification of sulfated glycosaminoglycans, visible in the right panel of Figure 5B, similarly revealed a significant decline in sGAG synthesis with increasing matrix elasticity and decreasing cell viability. As an example, values in PEG-dextran hydrogels with TGF-β3 stimulated chondrocytes declined from 1.14 ± 0.14 μg sGAG/μg DNA in 1 kPa hydrogels to 0.50 ± 0.19 μg sGAG/μg DNA in 15 kPa hydrogels down to 0.18 ± 0.04 μg sGAG/μg DNA in 30 kPa hydrogels. In contrast to observations in fibrin and silk/fibrin hydrogels, where DNA content, depicted in Supplementary Figure S6, increased twofold in all hydrogels upon growth factor stimulation, TGF-β3 failed to elicit proliferative activity in PEG-dextran hydrogels as corroborated by matching DNA quantities in both unstimulated and stimulated cultures.

## Conclusion

This study was aimed at elucidating the effect of matrix elasticity on the redifferentiation of primary human chondrocytes to obtain an optimized *in vitro* model for articular cartilage trauma and disease. Three well known hydrogels, fibrin, silk/fibrin and PEG-dextran were selected based on stiffness-tunability and characterized using oscillatory rheology. Hydrogel composition was adjusted to achieve defined matrix elasticities representing the stiffness of a wide range of bodily tissues including the elasticity of the perichondral space (*E* = 30 kPa). Rheological assessment revealed the suitability of both fibrin and PEG-dextran hydrogels for fine-tuning of matrix elasticity up to 30 kPa Young’s modulus. Results of this study clearly demonstrate the superior chondrogenic properties of pure fibrin over both silk-modified fibrin and PEG-dextran. In more detail, fibrin hydrogel supported high cell viabilities and chondrotypic behavior with increasing matrix stiffness associated with the hallmarks of redifferentiation, spherical morphologies, and especially matrix synthesis (Caron et al., 2012). Matrices mimicking the perichondral space guided chondrocytes not only to form small halos, resembling native cartilage chondrons, but also to generate a thick cartilaginous matrix composed of sGAGs and collagen type II. In other words, chondrocytes cultivated in pure fibrin hydrogels embedded themselves within a native ECM-enriched microenvironment under physiologic matrix elasticity, especially upon stimulation with TGF-β3. Additional beneficial effects of perichondral space-like matrix elasticity were further corroborated by elevated redifferentiation indices of collagen type II versus collagen type I and sGAG synthesis. Although the addition of 25% silk fibroin, intended to support chondrogenic differentiation, showed high viability and increased chondrotypic morphology with increasing matrix stiffness, it adversely affected redifferentiation capacity of embedded chondrocytes. As a result, we do not recommend the addition of silk fibroin to improve chondrogenic behavior of primary chondrocytes without additional assessment. In the case of PEG-dextran, physiologic morphologies were induced irrespective of matrix elasticity, but chondrocytes failed to remain viable especially in matrices of high stiffness, probably due to pore sizes below 10 nm restricting cell motility, proliferation and nutrient supply. Although dextran-based hydrogels generally seem to support chondrogenic redifferentiation based on elevated aggrecan gene expression, cell vitality becomes the key limiting factor.

In summary, we report the interplay between biomechanical cues, matrix composition and biochemical factors as a key aspect modulating chondrogenic phenotype. Only redifferentiated chondrocytes are capable of generating an *in vitro* cartilage analog, which is crucial for reliably generating functional tissue models of the synovial joint. In the last decade, conflicting information on the effect of matrix stiffness on chondrocytic behavior has been published and reliable knowledge of 3D biomechanical cues is still in its infancy. Consequently, our detailed study represents an important step in elucidating physiologically relevant matrix elasticity on cellular behavior.

## Data Availability Statement

The raw data supporting the conclusions of this article will be made available by the authors, without undue reservation, to any qualified researcher.

## Author Contributions

BB, SN, and PE designed the study. BB and SS executed experiments, performed RT-qPCR and sGAG quantification and analyzed the data. BS conducted histological sectioning and staining of the specimens. BB, SS, AT, SN, and HR interpreted the data. BB, SS, and PE drafted the manuscript with section contributions from BS, SN, and AT. All authors revised and approved the final manuscript.

## Conflict of Interest

The authors declare that the research was conducted in the absence of any commercial or financial relationships that could be construed as a potential conflict of interest.

## Supplementary Material

FIGURE S1 Loss, storage, and complex modulus as well as Young’s Modulus development of PEG-dextran hydrogels during gelation (*n* = 3). Frequency sweep and strain sweep of polymerized PEG-dextran hydrogels for the adequate selection of rheological parameters.

FIGURE S2 Loss modulus, storage, and complex modulus as well as Young’s Modulus development of fibrin hydrogels during gelation (*n* = 3).

FIGURE S3 Viability of primary chondrocytes determined by Live (Calein-AM)/Dead (Ethidium-H1) staining in **(A)** fibrin, **(B)** silk fibrin and **(C)** PEG-dextran hydrogels of 1 kPa, 15 kPa and 30 kPa elasticity without and with addition of TGF-β3. Pictures show epifluorescence images of whole hydrogel clots. (Scale bar: 100 μm).

FIGURE S4 Histological images of primary chondrocytes in PEG-dextran hydrogels of 1 kPa, 15 kPa, and 30 kPa elasticity stained with **(A)** H & E for morphology, **(B)** alcian blue for sGAG and **(C)** collagen type II antibodies. (Scale bar: 50 μm).

FIGURE S5 Gene expression of collagen type I and collagen type II in fibrin, silk/fibrin and PEG-dextran hydrogels of different elasticities. Depicted as mean with SEM (*n* = 9). ^∗^*p* < 0.1; ^∗∗^*p* < 0.05; ^***^*p* < 0.01; ^**#x002A;*^*p* < 0.001.

FIGURE S6 Quantification of total sGAG and total DNA amount per hydrogel for fibrin, silk/fibrin and PEG-dextran. Depicted as mean with SEM (*n* = 9). ^∗^*p* < 0.1; ^∗∗^*p* < 0.05; ^***^*p* < 0.01; ^****^*p* < 0.001.
