# Supplementary figures and images for: Stiffness Matters: Fine-Tuned Hydrogel Elasticity Alters Chondrogenic Redifferentiation
Source: Front Bioeng Biotechnol. 2020 Apr 30;8:373. doi: 10.3389/fbioe.2020.00373 (PMC7204401; doi:10.3389/fbioe.2020.00373)

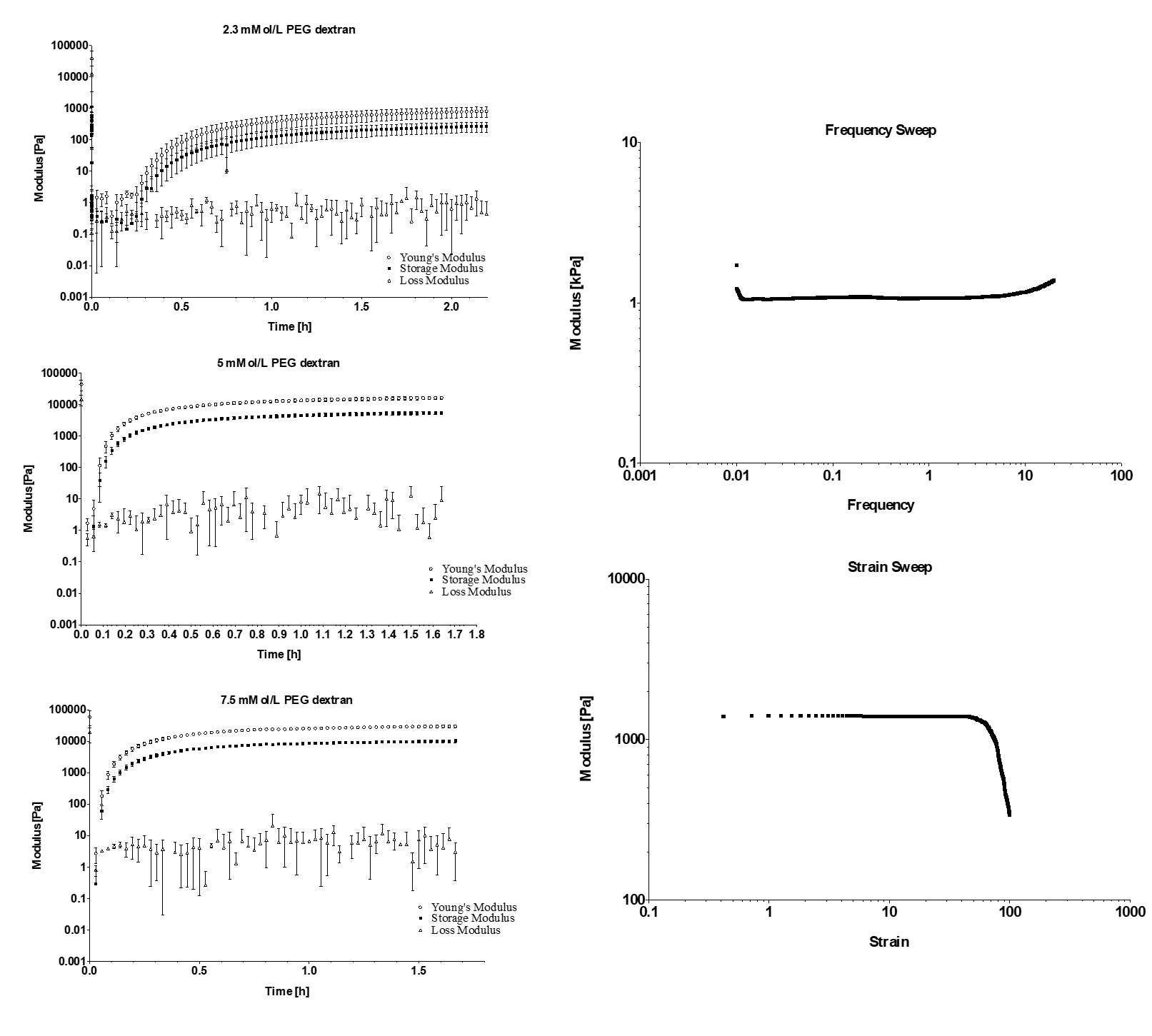

Supplement: FIGURE S1 — Loss, storage, and complex modulus as well as Young’s Modulus development of PEG-dextran hydrogels during gelation (n = 3). Frequency sweep and strain sweep of polymerized PEG-dextran hydrogels for the adequate selection of rheological parameters. [file Image_1.TIF]

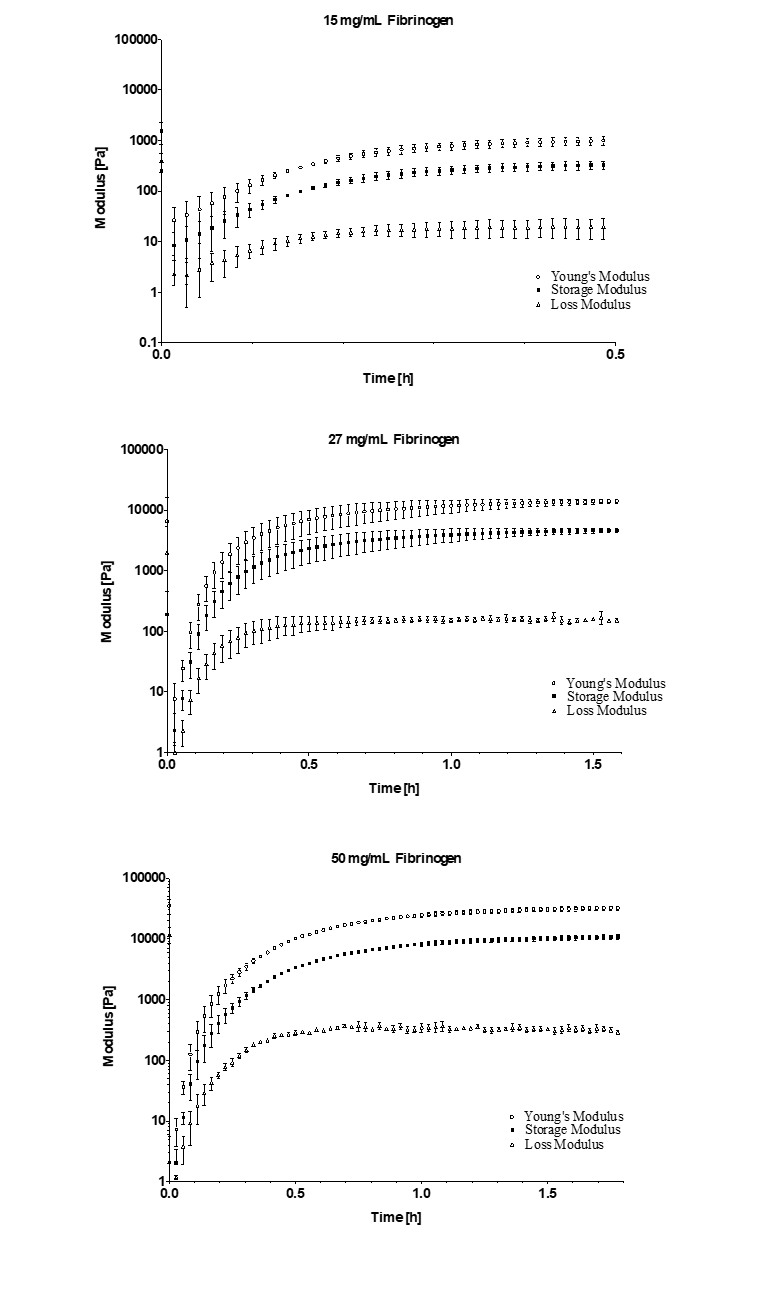

Supplement: FIGURE S2 — Loss modulus, storage, and complex modulus as well as Young’s Modulus development of fibrin hydrogels during gelation (n = 3). [file Image_2.TIF]

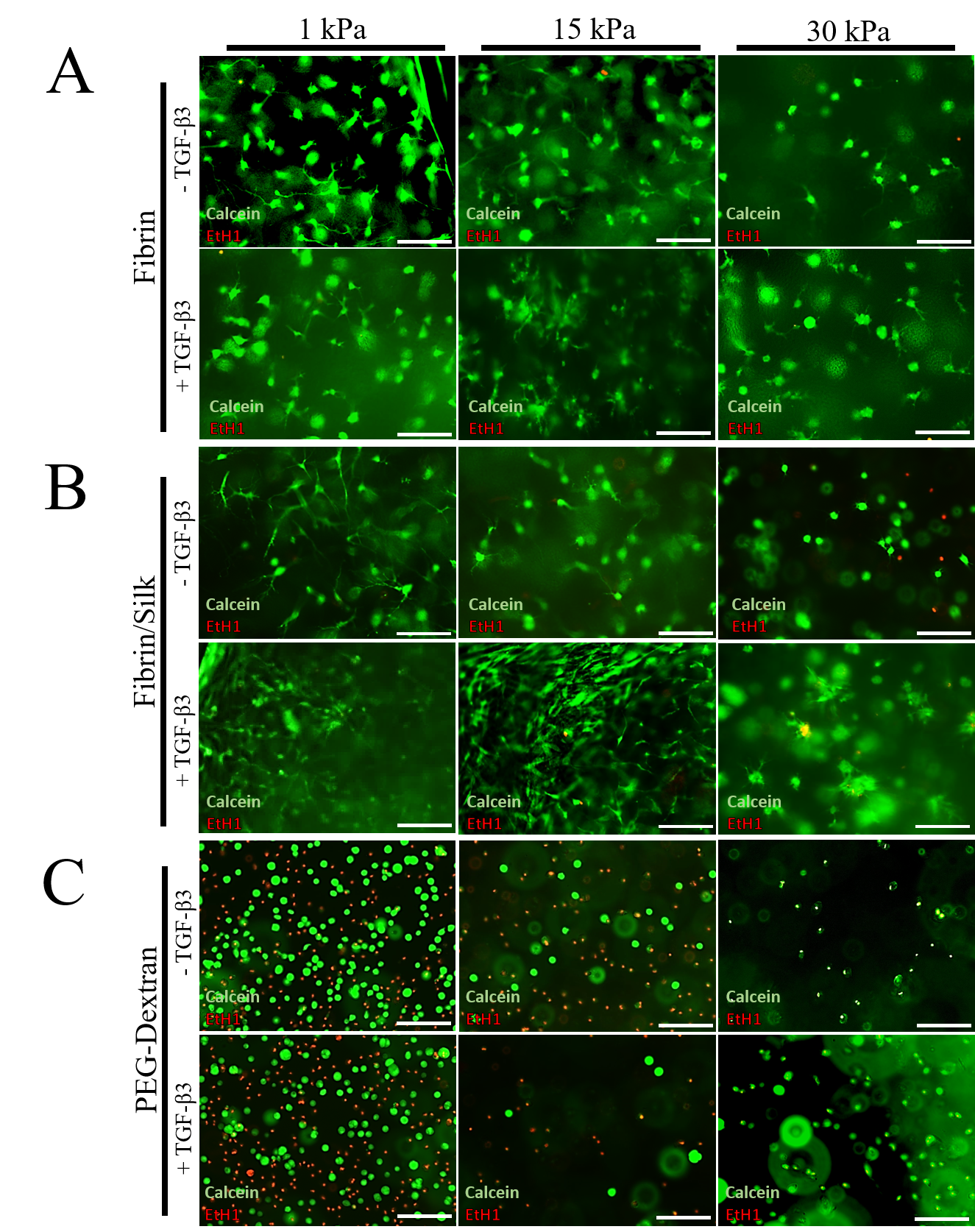

Supplement: FIGURE S3 — Viability of primary chondrocytes determined by Live (Calein-AM)/Dead (Ethidium-H1) staining in (A) fibrin, (B) silk fibrin and (C) PEG-dextran hydrogels of 1 kPa, 15 kPa and 30 kPa elasticity without and with addition of TGF-β3. Pictures show epifluorescence images of whole hydrogel clots. (Scale bar: 100 μm). [file Image_3.TIF]

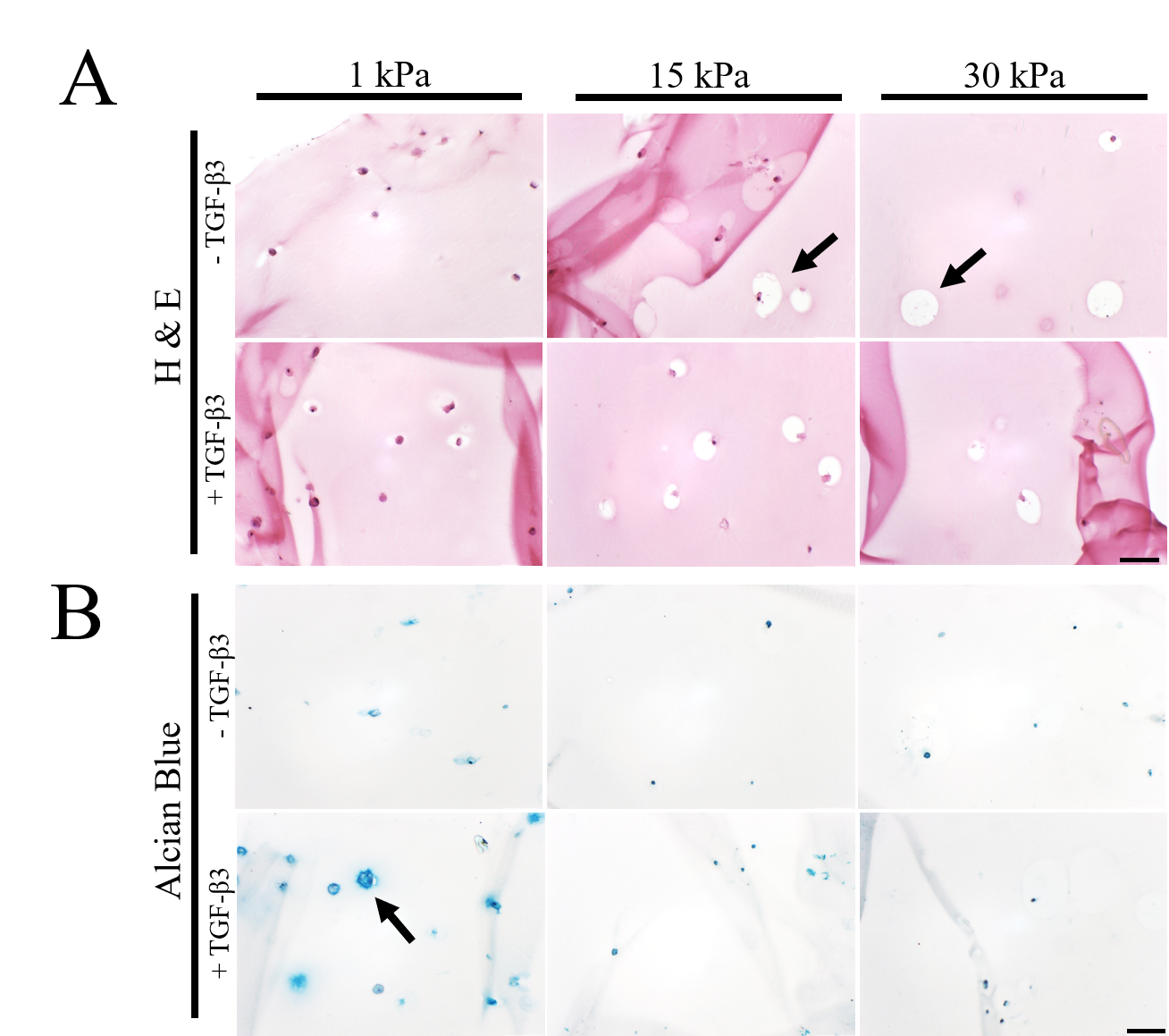

Supplement: FIGURE S4 — Histological images of primary chondrocytes in PEG-dextran hydrogels of 1 kPa, 15 kPa, and 30 kPa elasticity stained with (A) H & E for morphology, (B) alcian blue for sGAG and (C) collagen type II antibodies. (Scale bar: 50 μm). [file Image_4.TIF]

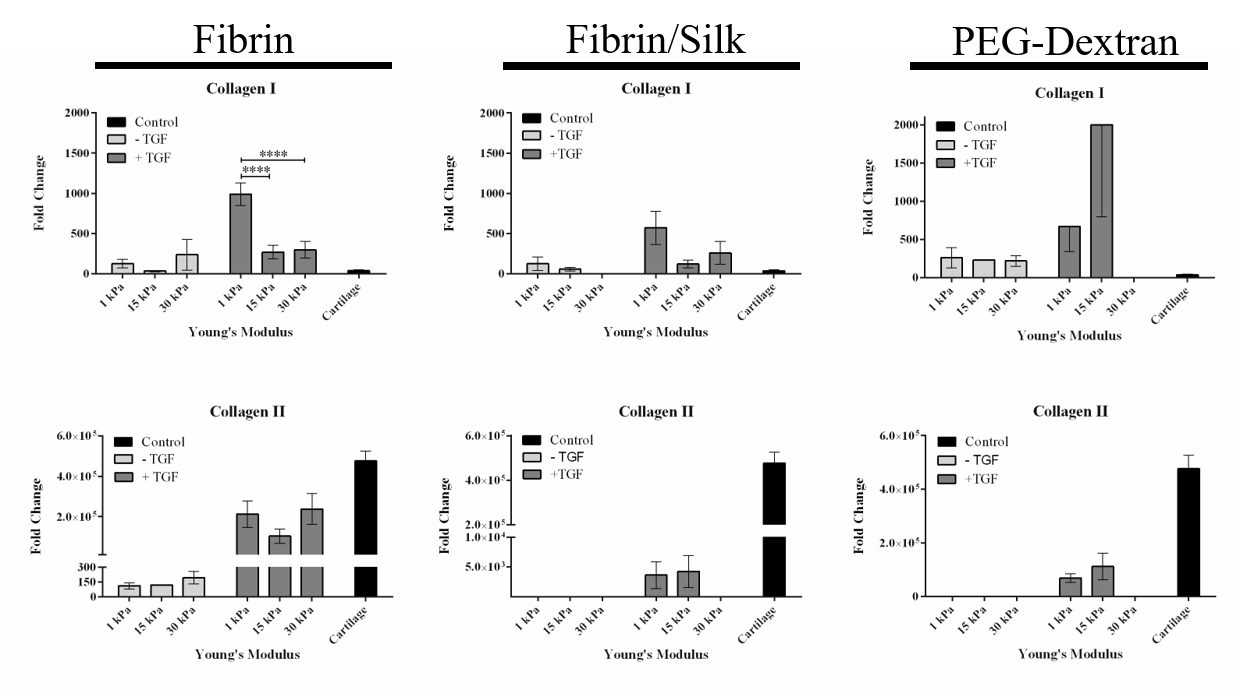

Supplement: FIGURE S5 — Gene expression of collagen type I and collagen type II in fibrin, silk/fibrin and PEG-dextran hydrogels of different elasticities. Depicted as mean with SEM (n = 9). ∗p < 0.1; ∗∗p < 0.05; ***p < 0.01; **#x002A;*p < 0.001. [file Image_5.TIF]

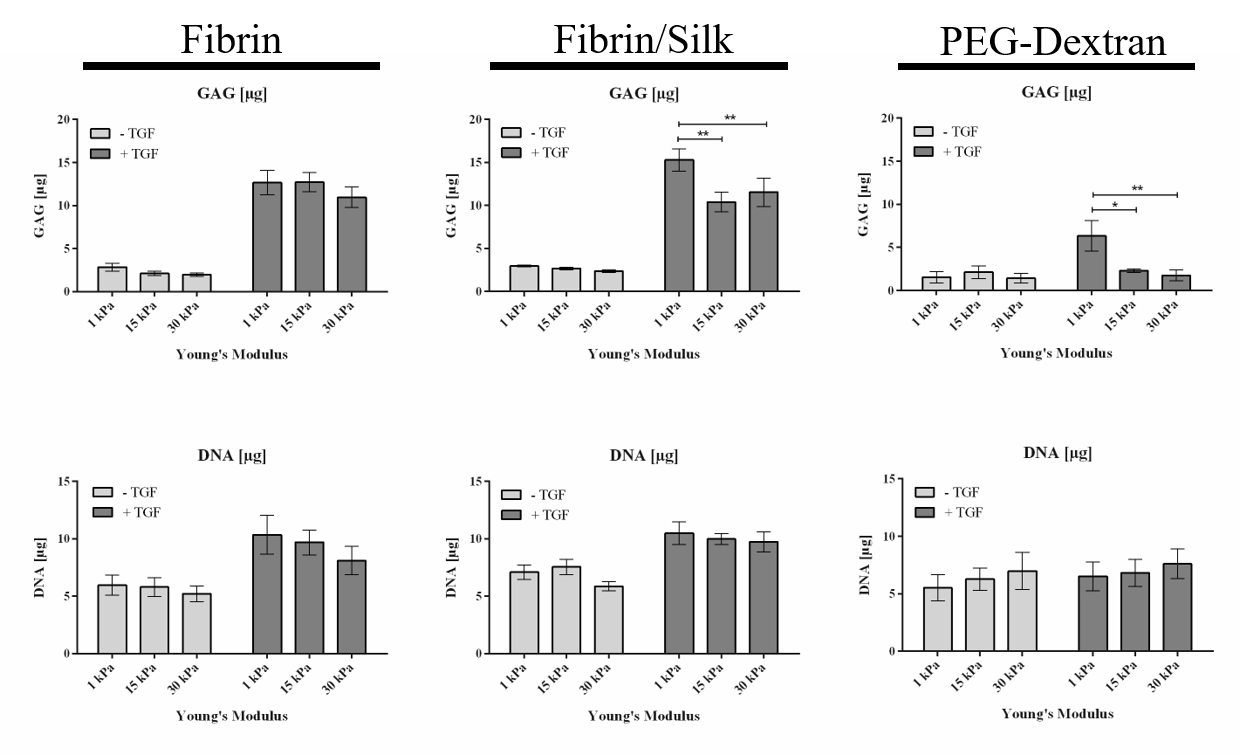

Supplement: FIGURE S6 — Quantification of total sGAG and total DNA amount per hydrogel for fibrin, silk/fibrin and PEG-dextran. Depicted as mean with SEM (n = 9). ∗p < 0.1; ∗∗p < 0.05; ***p < 0.01; ****p < 0.001. [file Image_6.TIF]
